# Evaluation of Physical Activity and Lifestyle Interventions During and After Chemotherapy for Ovarian Cancer: A Systematic Review of Patient Experiences and Clinical Outcomes

**DOI:** 10.7759/cureus.110295

**Published:** 2026-06-05

**Authors:** Hafiza Ateeqa Mubarak, Aqsa Mandvia, Muhammad Hassan Younas, Saadia Hassan, Vidya Raman, Aisha Ayaz, Vaisnavy Govindasamy, Kaushalendra Mani Tripathi, Abdul Samed Sulemana, Muaz Shafique Ur Rehman

**Affiliations:** 1 Obstetrics and Gynaecology, Ghurki Trust Teaching Hospital, Lahore, PAK; 2 Obstetrics and Gynaecology, Gloucestershire Royal Hospital, Gloucester, GBR; 3 Internal Medicine, King Edward Medical University, Lahore, PAK; 4 Obstetrics and Gynaecology, Frimley Park Hospital, Frimley, GBR; 5 Obstetrics and Gynaecology, Noble’s Hospital, Braddan, IMN; 6 Obstetrics and Gynaecology, Frimley Health NHS Foundation Trust, Frimley, GBR; 7 Internal Medicine, South Tees Hospitals NHS Foundation Trust, Middlesbrough, GBR; 8 Internal Medicine, White River Health Medical Center, Batesville, USA; 9 Obstetrics and Gynaecology, Ghana College of Physicians and Surgeons, Accra, GHA; 10 Obstetrics and Gynaecology, Mampong Government Hospital, Mampong, GHA; 11 Community Medicine, Allama Iqbal Medical College, Lahore, PAK

**Keywords:** chemotherapy, gynecologic cancers, interventions, ovarian cancer, physical activity

## Abstract

Ovarian cancer is a highly lethal gynecological malignancy, with chemotherapy-associated toxicity leading to fatigue, reduced physical function, and poor quality of life. Physical activity and lifestyle interventions may offer supportive benefits, but evidence in ovarian cancer remains limited and heterogeneous. This study aimed to assess how lifestyle modifications and physical activity during and after chemotherapy affect clinical outcomes and patient-reported experiences in women with ovarian cancer.

A systematic review was conducted in accordance with PRISMA standards. PubMed, Embase, Scopus, and the Cochrane Library were searched from inception to May 2026. Methodological quality was evaluated using the Cochrane Risk of Bias tool (RoB 2.0) for randomized controlled trials and the Newcastle-Ottawa Scale (NOS) for cohort and prospective studies. Six studies, totaling 724 patients, were included. The interventions included aerobic, resistance, and multimodal exercise routines. Across studies, physical function (six-minute walk test (6MWT): 395-571 m), muscle strength, and mobility were maintained or enhanced. Fatigue levels improved consistently across validated scales in five studies. All studies found that quality of life increased or remained stable. In one study, exercise was linked to improved treatment tolerance, including appropriate chemotherapy dose intensity and lower neurotoxicity. Adherence (81%-92%) and retention (70%-100%) rates demonstrated high feasibility. Physical activity interventions during and after chemotherapy appear to be practical and effective in improving functional outcomes, fatigue, and quality of life in patients with ovarian cancer. More large-scale trials are required to standardize interventions and validate long-term benefits.

## Introduction and background

Late diagnosis and high recurrence rates result in ovarian cancer being one of the most aggressive gynecologic cancers and one of the largest health problems worldwide. Global cancer data show that more than 300,000 new cases of ovarian cancer and more than 200,000 deaths are caused by the disease annually, and this mortality is disproportionately high in low- and middle-income countries [1,2]. Standard treatment includes cytoreductive surgery and platinum-based chemotherapy. These therapies are useful in improving survival but also have significant treatment-associated toxicity and dysfunction. Because ovarian cancer is frequently diagnosed at an advanced stage and is characterized by high rates of recurrence, many patients experience prolonged treatment courses and persistent physical and psychosocial burdens that can negatively affect daily functioning and quality of life.

Chemotherapy patients often suffer from cancer-related fatigue, weakened muscles, decreased aerobic capacity, and a lower quality of life, all of which lead to lower tolerance to treatment and long-term incapacity [3]. Physical deconditioning brought on by extended periods of inactivity during therapy frequently exacerbates these negative consequences. Pharmacological symptom treatment and dose modification have historically been the main emphasis of supportive care measures; nevertheless, these methods fall short in addressing the underlying functional impairment.

As potential non-pharmacological methods to reduce treatment-related toxicity and enhance patient outcomes, physical activity interventions primarily involve structured aerobic, resistance, or flexibility exercises, and lifestyle interventions may additionally incorporate behavioral counseling, education, rehabilitation strategies, and other health-promoting practices [4]. Exercise may increase chemotherapy tolerance and lower hospitalization rates while also improving fatigue, physical function, and health-related quality of life, according to research from general oncology populations [5]. Several studies conducted in ovarian cancer populations have reported that exercise interventions are feasible and well-tolerated during and after treatment, with potential benefits for physical functioning, fatigue, psychological well-being, and overall quality of life. However, these studies are often limited by small sample sizes, heterogeneous intervention protocols, differences in treatment settings, and variability in outcome assessment, making it difficult to draw definitive conclusions regarding their clinical effectiveness.

Ovarian cancer-specific information remains scarce and inconsistent, with variation in exercise duration, intensity, and outcome measures despite growing interest in the field. Additionally, uneven reporting of patient-centered outcomes such as functional recovery, treatment feasibility, and fatigue experienced during active chemotherapy limits the use of these findings in clinical practice [6-8]. Furthermore, although exercise has been widely studied across several cancer types, evidence syntheses focused specifically on ovarian cancer patients receiving chemotherapy remain limited. Consequently, the extent to which physical activity interventions improve treatment-related outcomes and patient-reported experiences in this population has not been comprehensively established.

Thus, with an emphasis on clinical outcomes and patient-reported experiences, this systematic review attempts to assess the impact of physical exercise and lifestyle interventions during and after chemotherapy in ovarian cancer. In general, integrating rehabilitation into conventional oncologic therapy pathways requires defining the function of organized exercise in ovarian cancer care.

## Review

Methods

The Preferred Reporting Items for Systematic Reviews and Meta-Analyses (PRISMA) criteria were followed in conducting this systematic review (CRD420261392801) [9].

Study Selection Process and Search Strategy

To assess the impact of physical exercise and lifestyle modifications both during and after chemotherapy in patients with ovarian cancer, we created a thorough and organized search strategy. A comprehensive search of the PubMed, Cochrane Library, Embase, and Scopus databases was conducted from inception through May 2026 until all relevant studies were identified. Medical Subject Headings (MeSH) and free-text keywords were combined using an “in-text” search strategy. “Ovarian cancer,” “physical activity,” “exercise,” “lifestyle intervention,” “chemotherapy,” “rehabilitation,” “fatigue,” and “quality of life,” along with their synonyms, were the main search terms. Supplementary search details are provided in Appendix 1.

To ensure reproducibility and transparency, a systematic screening process was used. First, titles and abstracts were assessed for eligibility by two independent reviewers (Aqsa Mandvia and Muhammad Hassan Younas). All potentially eligible studies were then subjected to full-text screening. A third reviewer was consulted to resolve any disagreements between the reviewers.

Eligibility Criteria

Studies that met the following criteria were included: adult females with ovarian cancer receiving or having received chemotherapy; any structured exercise regimen or lifestyle modification (including walking, resistance, aerobic, or multimodal exercise); at least one clinical outcome (e.g., physical function, strength, fatigue, quality of life, treatment tolerance, or body composition); and prospective cohort studies, randomized controlled trials, or feasibility studies.

The exclusion criteria were: studies using animal models; conference abstracts; reviews; editorials; case reports without primary data; studies that did not include physical activity or exercise as the main intervention; and non-English language articles.

Data Extraction

Data were obtained independently using a common Microsoft Excel (Microsoft® Corp., Redmond, WA, USA) template to ensure uniformity. The following variables were extracted: author information, publication year, study design, sample size, study characteristics, intervention type, follow-up duration, comparator groups, and outcome measures. The primary outcomes were physical function (six-minute walk test (6MWT)), muscle strength, and cancer-related fatigue. Secondary outcomes were quality of life, chemotherapeutic tolerance, neurotoxicity, body composition, adherence, and retention. Data are presented as odds ratios, event/total, median (range/IQR), and mean ± standard deviation, as appropriate.

Data Synthesis

A quantitative meta-analysis was not performed because the included studies exhibited substantial clinical and methodological heterogeneity. Specifically, considerable differences existed in study design, patient populations, intervention characteristics (type, intensity, frequency, and duration of exercise), treatment timing, outcome definitions, assessment tools, and reporting methods. Consequently, the outcomes were not considered sufficiently comparable for statistical pooling.

Therefore, a narrative synthesis approach was employed. Extracted data were summarized descriptively and organized according to predefined outcome domains, including fatigue, quality of life, physical function, muscle strength, feasibility and adherence, body composition, chemotherapy completion, and treatment-related adverse effects. Findings were compared across studies, with emphasis placed on the consistency, direction, and clinical relevance of the reported effects.

Quality Assessment

According to the study design, validated assessment tools were used to evaluate the methodological quality of the included studies. The Newcastle-Ottawa Scale (NOS), which has a maximum score of 9 stars and evaluates three methodological domains: selection (four items), comparability (one item), and outcome assessment (three items), was used to evaluate prospective and cohort studies. Studies were classified as having a low, moderate, or high risk of bias based on their overall scores (9) [10].

**Figure 1:**
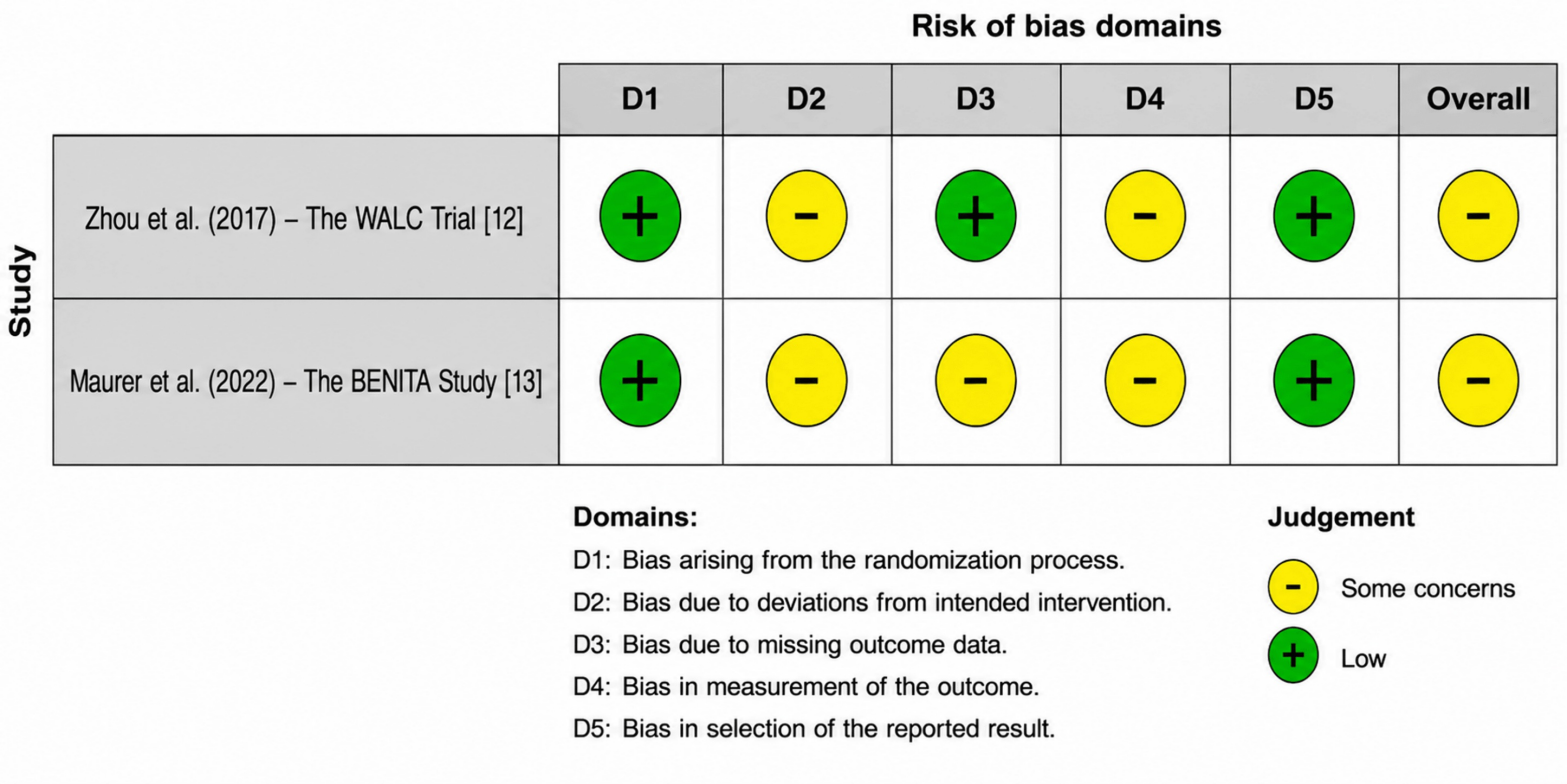
Risk of bias assessment of included studies in domains This figure presents the risk of bias evaluation of the included studies using the Cochrane Risk of Bias (RoB 2) tool across five domains: bias arising from the randomization process (D1), bias due to deviations from intended interventions (D2), bias due to missing outcome data (D3), bias in measurement of outcomes (D4), and bias in selection of the reported result (D5). Both included studies demonstrated a low risk of bias in domains related to randomization and selection of reported results, while some concerns were observed mainly in deviations from intended interventions, outcome measurement, and overall risk of bias assessment. No domain was judged to be at high risk of bias.

**Figure 2:**
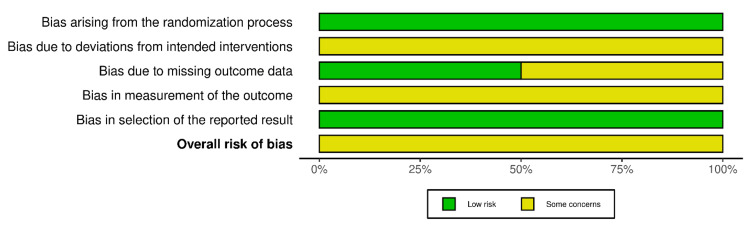
Traffic plots of included studies This figure illustrates the proportion of studies categorized as low risk and some concerns across each methodological domain of the Cochrane RoB 2 tool. All studies demonstrated low risk of bias for the randomization process and selection of reported results, whereas some concerns were identified in deviations from intended interventions and measurement of outcomes. Missing outcome data showed a mixed distribution of low risk and some concerns. No high-risk judgments were identified across any domain.

The Cochrane Risk of Bias Tool version 2.0 (RoB 2.0), which evaluates bias across five domains - randomization process, deviations from intended interventions, missing outcome data, outcome measurement, and selection of reported results - was used to evaluate randomized controlled trials. According to RoB 2.0 guidelines, each study was categorized as having low risk of bias, some concerns, or high risk of bias (Figures 1-2) [11].

Two reviewers (Saadia Hassan and Vidya Raman) independently assessed the quality, and any differences were resolved through consultation with a third reviewer. A summary of the quality evaluation results for each of the included studies is provided [11].

Ethical Considerations

The study did not require ethical approval because it was based on already published literature. All data were gathered from publicly available sources, and no patient-specific information was accessed or evaluated.

Results

Study Selection Process

**Figure 3:**
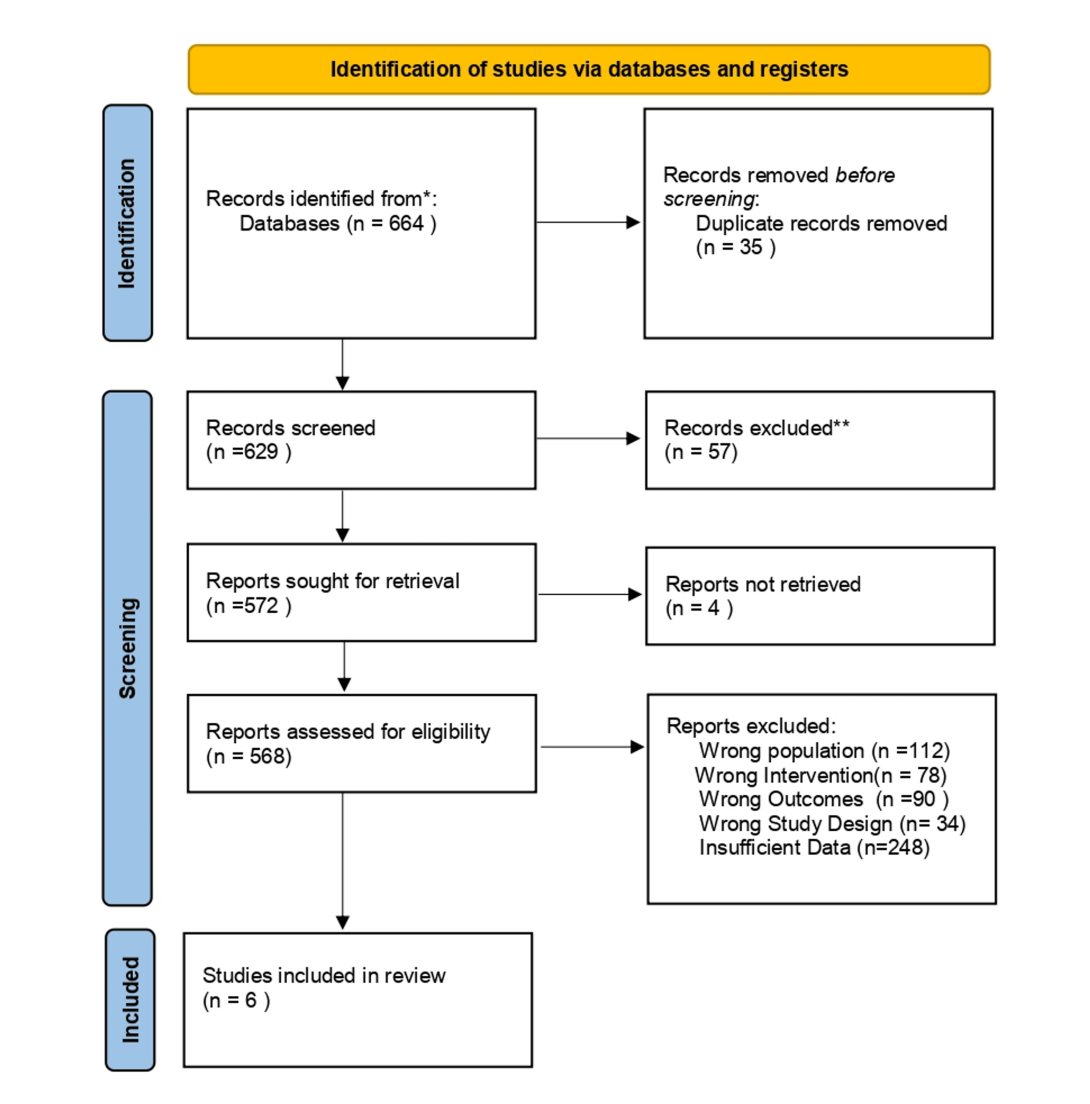
PRISMA flow diagram of included studies for study selection process PRISMA flow diagram illustrating the process of study identification, screening, eligibility assessment, and inclusion. A total of 664 records were identified through database searching, with six studies ultimately included in the final review after removal of duplicates and exclusion of ineligible reports.

A total of 664 records were identified through database searches, with 35 duplicates removed, leaving 629 studies for screening. Following title and abstract screening, 57 records were excluded, and 572 reports were sought for retrieval; four were unavailable. A total of 568 full-text articles were assessed for eligibility, with exclusions due to incorrect population (n = 112), intervention (n = 78), outcomes (n = 90), study design (n = 34), and inadequate data (n = 248). Finally, six studies met the inclusion criteria and were included in the systematic review, as shown in Figure 3.

Baseline Characteristics and Patient Demography

This systematic review comprised six studies with 724 patients, including two randomized controlled trials, two single-arm feasibility studies, one non-randomized phase II pilot trial, and one nationwide cohort study. The sample sizes ranged from 15 to 503. The mean/median age across the trials was fairly consistent (57.3-64 years). BMI ranged from 25.3 to 29.0 kg/m², with many studies reporting a significant proportion of normal-weight participants. Most patients had advanced-stage (III/IV) ovarian cancer (approximately 55%-100%), and the majority were receiving post-diagnosis treatment, primarily adjuvant chemotherapy, with some cohorts including recurrent or post-first-line survivors. Overall, the group consisted primarily of older women with advanced epithelial ovarian cancer who were currently undergoing or had recently completed systemic therapy, as shown in Table 1.

**Table 1:** Study characteristics and patient’s demography Studies included: [12-17]. NR: Not reported; BMI: Body Mass Index; OC: Ovarian Cancer; KPS: Karnofsky Performance Status; ECOG: Eastern Cooperative Oncology Group performance status; MET: Metabolic Equivalent of Task; RCT: Randomized Controlled Trial; Adjuvant therapy: Treatment given after primary treatment (e.g., surgery); Neoadjuvant therapy: Treatment given before primary treatment.

Study (Lead Author)	Study Design	N	Age	BMI (kg/m²)	Stage III/IV (%)	Treatment Phase	Histology	Activity Level	Performance Status	Marital Status	Location
Zhou et al. (2017) (WALC) [12]	Randomized controlled trial	144	57.3 ± 8.6 years	29.0 ± 7.0	54.9%	93.1% received prior chemotherapy	NR	28.3 ± 41.6 min/week	NR	72.9% married/partnered	Connecticut, USA
Maurer et al. (2022) (BENITA) [13]	1:1 randomized controlled trial	15	58 (21-77) years	60% normal weight	73.3%	80% adjuvant; 20% neoadjuvant	NR	60% 0-4 MET h/week	ECOG < 2 (eligibility)	NR	Hamburg, Germany
Newton et al. (2011) [14]	Non-randomized phase II pilot trial	17	60 ± 8 years	40% healthy weight	88%	82% adjuvant; 18% neoadjuvant	82% serous	NR	NR	58% de facto/married	Brisbane, Australia
Mizrahi et al. (2015) [15]	Prospective single-arm feasibility study	30	58.6 ± 10.9 years	25.3 ± 7.1	80%	100% recurrent (2nd/3rd line)	100% epithelial OC	Insufficiently active	KPS 74.5 ± 10.1	NR	Sydney/Canberra, Australia
Schofield et al. (2023) [16]	Prospective single-arm pragmatic study	15	64 (33-72) years	26.8 ± 6.3	100%	100% post-first-line survivors	100% epithelial OC	NR (exercise-excluded structured program)	NR	73.3% partnered	Perth, Australia
Ross et al. (2024) (OPAL) [17]	National prospective cohort study	503	60.3 ± 10.5 years	46% < 25 kg/m²	74%	73% adjuvant; 22% neoadjuvant	78% high-grade serous	52% minimal exercise	NR	NR	Australia (national)

Quality Assessment

Zhou et al. (2017) [12] and Maurer et al. (2022) [13] were both judged to be of low risk in D1 (bias due to the randomized procedure), indicating appropriate use of randomization techniques. Both investigations raised concerns about bias due to deviations from intended interventions (D2), predominantly due to limited information on blinding and adherence. Zhou et al. (2017) reported a low risk of bias in D3 (missing outcome data), whereas Maurer et al. (2022) expressed some concerns about insufficient reporting of outcome data. Both studies had some concerns in D4 (bias in outcome measurement), due to the possibility of detection bias and insufficient information on blinding. For D5 (bias in the selection of the reported result), both trials were considered low risk, indicating agreement between reported and prespecified outcomes. The overall risk of bias in both studies was considered “some concerns,” with no study classified as having “high risk” (Figures 1-2).

Additionally, for the other included studies, the methodological quality of the cohort and prospective studies was moderate to high on the NOS. Three studies [14-16] were rated as having moderate quality (5/9) and had the majority of weaknesses in the comparability and outcome assessment domains. By contrast, Ross et al. (2024) [17] scored a perfect 9/9, indicating good methodological quality in design, follow-up, and outcome assessment. Most studies were of moderate risk of bias, with one high-quality cohort study adding to the evidence base (Table 2).

**Table 2:** Newcastle-Ottawa quality assessment scale of included cohort studies ☆: Indicates compliance with the criteria. The numbers in parentheses represent the maximum possible score for each criterion. The overall score reflects the quality assessment of each study. A score of up to 9 indicates high quality; a score of 6 or above indicates good quality; and a score below 6 indicates low quality. ★: The Newcastle-Ottawa Scale stars indicate study quality and risk of bias. A study scoring 9/9 stars demonstrates higher methodological compliance and lower risk of bias, whereas studies with fewer than 6 stars indicate lower compliance and a higher potential for bias. Studies included: [14-17].

Study ID	Representativeness of the exposed cohort (1)	Selection of the non-exposed cohort (1)	Ascertainment of exposure (1)	Demonstration that outcome not present at start (1)	Comparability of cohorts on basis of design/analysis (2)	Assessment of outcome (1)	Was follow-up long enough (1)	Adequacy of follow-up of cohorts (1)	Total score (9)
Newton et al. (2011) [14]	★	☆	★	★	☆☆	☆	★	★	5/9
Mizrahi et al. (2015) [15]	★	☆	★	★	☆☆	☆	★	★	5/9
Schofield et al. (2023) [16]	★	☆	★	★	☆☆	☆	★	★	5/9
Ross et al. (2024) [17]	★	★	★	★	★★	★	★	★	9/9

Outcomes

This extensive review included six main studies with 724 patients, of whom 257 were classified as higher-intensity exercisers or received structured exercise therapies. The studies included multimodal rehabilitation programs, walking-based therapies, resistance training, and aerobic exercise delivered during or around chemotherapy treatment for ovarian cancer. Some studies investigated outcomes including physical function, muscle strength, fatigue, quality of life, treatment tolerability, neurotoxicity, body composition, and feasibility-related measures. A detailed summary of the clinical, functional, and feasibility outcomes reported across the included studies is presented in Table 3.

**Table 3:** Clinical and feasibility outcomes for included studies Studies included: [12-17]. 6MWT: Six-Minute Walk Test; CI: Confidence Interval; EORTC QLQ-C30: European Organisation for Research and Treatment of Cancer Quality of Life Questionnaire-Core 30; FACT-F: Functional Assessment of Cancer Therapy-Fatigue; FACT-O: Functional Assessment of Cancer Therapy-Ovarian; FACT-TOI: Functional Assessment of Cancer Therapy-Trial Outcome Index; FACIT-F: Functional Assessment of Chronic Illness Therapy-Fatigue; HRQOL: Health-Related Quality of Life; MCS: Mental Component Summary; MFI-20: Multidimensional Fatigue Inventory-20; NR: Not Reported; OR: Odds Ratio; PCS: Physical Component Summary; RDI: Relative Dose Intensity; SF-36: 36-Item Short Form Health Survey; SPHERE: Somatic and Psychological Health Report; TUG: Timed Up and Go.

Study	Physical function/aerobic capacity	Muscle strength/mass	Fatigue	Quality of life/HRQOL	Treatment tolerance	Other outcomes	Adherence/retention
Newton et al. (2011) [14]	6MWT: 395 m (356-460)	NR	NR	FACT-O: 113 (67-148)	≥85% RDI: 13/17 (76%)	NR	82% adherence; 100% retention
Zhou et al. (WALC) (2017) [12]	NR	NR	FACT-F: +4.0 ± 1.1	SF-36 PCS: +1.8 ± 1.1; SF-36 MCS: +1.6 (95% CI -0.6 to 3.9)	NR	NR	166.0 min/week activity; 78.5% retention
Mizrahi et al. (2015) [15]	VO₂ cycle: 24.9 ± 6.8	Upper body: 25.8 ± 5.5 kg; Lower body: 35.9 ± 11.8 kg	SPHERE: 1.2 ± 1.4	FACT-O: 109.9 ± 21.0	Mean RDI: 94.6%	Balance: 47.1 ± 28.6 s	81% adherence; 70% retention
Maurer et al. (BENITA) (2022) [13]	6MWT: 570.7 m	Handgrip: 24.8 kg	MFI-20 Physical: 7.0	Global Health Status: 70.8	NR	NR	83.7% adherence; 73.3% retention
Schofield et al. (2023) [16]	400-m walk: 282.7 ± 60.6 s	Chest press: 22.7 ± 5.6 kg; Lean mass: 40.7 ± 6.2 kg; Leg press: 95.5 ± 39.5 kg	EORTC Fatigue: 27.8 ± 11.3	EORTC QLQ-C30 Social Function: 79.8 ± 14.9	NR	TUG mobility: 7.2 ± 1.5 s	92% adherence; 100% retention
Ross et al. (OPAL) (2024) [17]	NR	NR	FACIT-F: 32.2 (95% CI 27.9-36.5)	FACT-TOI: 69.4 (95% CI 64.1-74.7)	≥85% RDI OR: 1.70 (95% CI 0.9-3.2)	Neurotoxicity OR: 0.50 (95% CI 0.29-0.88)	NR

Primary Outcomes

Physical function and aerobic capacity: Four studies reported aerobic capacity and physical function outcomes, including the 400 m walk test or the 6MWT. Newton et al. (2011) [14] reported an average 6MWD of approximately 395 ± 26 m following a walking intervention and chemotherapy. Following multimodal therapy, Maurer et al. (2022) [13] (BENITA study) reported a median post-intervention 6MWD of 570.7 m. Functional mobility was assessed using the 400 m walk test by Schofield et al. (2023) [16], and their results indicated a mean walk time of 282.7 ± 60.6 seconds following resistance training. A post-intervention VO₂-related value of 24.9 ± 6.8 was reported by Mizrahi et al. (2015) [15], who assessed aerobic performance using cycle ergometry. Overall, exercise had positive effects on physical function in three trials.

Upper and lower body strength: Maurer et al. (2022) [13] reported a median upper-extremity handgrip strength of 24.8 kg, indicating intact muscle function. In summary, all three trials reporting strength-related outcomes after exercise showed improvements and/or maintenance in muscle performance, as shown by mean leg press strength (95.5 ± 39.5 kg), mean chest press strength (22.7 ± 5.6 kg), and mean peak knee extension force (8.8 ± 3.2 kg). Three studies reported results on muscular strength. After resistance training, the mean strength in the leg press and seated row was 35.9 ± 11.8 kg and 25.8 ± 5.5 kg, respectively, as reported by Mizrahi et al. (2015) [15].

Fatigue outcomes: Five studies used the FACT-F, FACIT-Fatigue, SPHERE, MFI-20, or EORTC fatigue scales to assess fatigue-related outcomes. Mizrahi et al. (2015) [15] reported low fatigue scores on the SPHERE instrument at post-intervention (1.2 ± 1.4), whereas Maurer et al. (2022) [13] reported low scores on the MFI-20 physical fatigue scale (median 7.0). Similarly, the mean FACIT-Fatigue score among higher-intensity exercisers was 32.2 (95% CI 27.9-36.5), suggesting a lower fatigue burden. Additionally, Schofield et al. (2023) [16] reported a mean EORTC tiredness score of 27.8 ± 11.3 after engaging in exercise. In cancer-related fatigue, FACT scores increased by 4.0 ± 1.1 after the intervention [12]. Overall, all five exercise interventions yielded good results regarding fatigue.

Quality of life outcomes: All six studies used the FACT-O, FACT-TOI, SF-36, EORTC QLQ-C30, or Global Health Status scales to report quality of life outcomes. Following the walking intervention, Newton et al. (2011) [14] reported a post-intervention FACT-O score of about 109.3 ± 20.3. Following exercise, Mizrahi et al. (2015) [15] found that the mean FACT-O global quality of life score was 109.9 ± 21.0. SF-36 physical component scores increased by +1.8 ± 1.1 in 2014. In contrast, mental component scores rose by 1.6 points (95% CI -0.6 to 3.9), according to Zhou et al. (2017) [12]. After receiving therapy, Maurer et al. (2022) [13] found that the median global health status score was 70.8. Additionally, following exercise, Schofield et al. (2023) [16] showed a mean EORTC QLQ-C30 social functioning score of 79.8 ± 14.9. According to Ross et al. (2024) [17], higher-intensity exercisers had a mean FACT-TOI score of 69.4 (95% CI 64.1-74.7). All six trials showed that quality of life outcomes were either maintained or enhanced after exercise treatments.

Secondary Outcomes

Neurotoxicity and treatment tolerance: Three studies reported on treatment tolerance and outcomes of chemotherapy completion. According to Newton et al. (2011) [14], 13 out of 17 participants (76%) attained a relative dose intensity (RDI) of at least 85%. According to Mizrahi et al. (2015) [15], treatment completers had an average RDI of 94.6% (range 75%-100%). Higher-intensity exercise involvement was associated with a lower risk of clinician-reported neurotoxicity according to Ross et al. (2024) [17] (OR 0.50, 95% CI 0.29-0.88). The same study also found a non-significant correlation (OR 1.70, 95% CI 0.90-3.20) between effective chemotherapy completion at ≥85% RDI and higher-intensity exercise. One study reported decreased neurotoxicity associated with exercise participation, whereas three trials overall reported positive treatment-tolerance outcomes.

Balance, mobility, and body composition: Two studies assessed mobility-related effects and balance. A mean post-intervention single-leg balance duration of 47.1 ± 28.6 seconds was observed by Mizrahi et al. (2015) [15], indicating enhanced postural stability. The Timed Up-and-Go (TUG) test was used by Schofield et al. (2023) [16] to measure mobility. They found that the mean completion time was 7.2 ± 1.5 seconds, with lower values indicating higher mobility and balancing performance.

One study reported results related to body composition. Following resistance training, Schofield et al. (2023) [16] showed a mean whole-body lean mass of 40.7 ± 6.2 kg, indicating that muscle mass was either preserved or improved during therapy.

Adherence and feasibility outcomes: Five trials provided adherence and feasibility outcomes, which were usually positive. During the walking intervention, Newton et al. (2011) [14] attained 100% retention and 82% adherence. Zhou et al. (2017) [12] showed a retention rate of 78.5% and an average weekly exercise participation of 166.0 minutes. Mizrahi et al. (2015) [15] reported 81% adherence and 70% retention under supervised exercise participation. According to Maurer et al. (2022) [13], retention and adherence rates were 73.3% and 83.7%, respectively. Over the course of the intervention, Schofield et al. (2023) [16] attained 100% retention and 92% adherence. Ross et al. (2024) [17] conducted observational OPAL cohort research but did not formally report feasibility findings. The viability of exercise treatments during ovarian cancer therapy was supported by overall adherence rates of 81% to 92% and retention rates of 70% to 100%.

Discussion

This systematic review suggests that structured physical activity and lifestyle interventions are feasible and may provide clinically meaningful benefits for patients with ovarian cancer undergoing or recovering from chemotherapy. The overall evidence indicates consistent improvements in fatigue, quality of life, physical function, and muscle strength, while also demonstrating high levels of intervention adherence and retention. These findings support the growing recognition of exercise as an important component of supportive oncology care and extend this evidence to the ovarian cancer population, for whom data remain comparatively limited.

Although much of the existing evidence originates from mixed-cancer populations, the consistency of benefits observed across ovarian cancer studies suggests that this patient group may derive similar functional and symptom-related improvements from structured exercise interventions. The findings of this systematic review align broadly with the wider exercise oncology literature while offering novel insights specific to ovarian cancer. A meta-analysis by Mustian et al. (2017) [18] found that exercise interventions reduce cancer-related fatigue with a moderate-to-large effect size (SMD = -0.30 to -0.80) across 44 randomized controlled trials. All five studies that assessed this domain showed consistent improvement in fatigue outcomes. Sweegers et al. (2018) [19] found that aerobic and resistance training improve global quality of life, physical well-being, and social functioning during active cancer treatment, supporting the findings of all six studies.

Improvements in physical function and aerobic capacity observed across the included studies are clinically meaningful because functional decline during chemotherapy is associated with increased symptom burden and reduced quality of life. The improvements in 6MWT performance reported in this review are consistent with findings from exercise interventions in patients with advanced cancer, where structured exercise programs were associated with improved physical functioning and exercise capacity [20]. Furthermore, preservation of muscle strength and lean body mass aligns with findings reported [20], who demonstrated favorable effects of exercise on body composition among women with ovarian cancer. Collectively, these findings suggest that exercise may help mitigate treatment-related deconditioning and maintain functional independence in this population.

Maintenance of muscle strength and physical performance may contribute to improved treatment tolerance and quality of life among women with ovarian cancer. Recent studies have reported that exercise interventions can attenuate treatment-related functional decline and support preservation of physical capacity during and after cancer therapy, findings that are consistent with the outcomes observed in this review [21,22].

Additionally, chemotherapy-induced peripheral neuropathy (CIPN) is one of the review's most innovative findings. Another study [23], which found that exercise, particularly balance and multimodal training, significantly reduces subjective and objective CIPN symptoms, supports the observed 50% reduction in neurotoxicity risk among higher-intensity exercisers (OR = 0.50) [23]. Exercise may protect against axonal damage caused by platinum-based drugs by upregulating neurotrophic factors (such as BDNF and NGF) and reducing systemic inflammation, according to mechanistic investigations. However, the findings of von Gruenigen et al. (2011) [24] in ovarian cancer survivors contradict the non-significant correlation between higher-intensity exercise and chemotherapy completion at ≥85% RDI; this disparity may be due to variations in baseline treatment adherence rates, regimen intensity, or the observational design of the contributing study. Notably, the observed reduction in chemotherapy-induced neurotoxicity represents one of the most clinically important findings of this review. Given that platinum-based chemotherapy remains a cornerstone of ovarian cancer treatment, interventions capable of mitigating neurotoxicity may improve both treatment tolerability and long-term survivorship outcomes. However, this finding was derived from a limited number of studies and should be interpreted cautiously until confirmed in larger prospective trials.

Collectively, these findings indicate that exercise interventions are not only effective but also practical and acceptable for patients with ovarian cancer. High adherence and retention rates suggest that structured exercise programs can be successfully integrated into supportive cancer care pathways, including home-based and remotely supervised models. Hence, the feasibility and adherence outcomes of our review showed that (81%-92% adherence, 70%-100% retention) are higher than the average rates for home-based exercise programs in oncology (60%-75% adherence, 65%-85% retention) reported by Kampshoff et al. (2014) [25]. This implies that people with ovarian cancer might be especially driven to participate in organized exercise. Because sarcopenia affects up to 40% of ovarian cancer survivors and is independently linked to lower survival, body composition results demonstrating preserved whole-body lean mass are clinically significant. Resistance training can increase lean mass by 1.2-2.0 kg over 12 weeks. Moreover, the trial by Johns et al. (2020) [21], which showed that combined resistance and balance training lowers fall risk by roughly 34% in cancer survivors with residual neuropathy, is consistent with the favorable balance and mobility outcomes (e.g., single-leg stance 47.1 s, TUG 7.2 s). Taken together, these comparisons highlight the internal validity of the current findings and their external generalizability to the broader body of evidence supporting exercise in cancer populations [26].

Limitations and strength

This review has several strengths. To our knowledge, it is among the first systematic reviews specifically evaluating physical activity and lifestyle interventions in ovarian cancer patients receiving or completing chemotherapy. The review incorporated a broad range of patient-reported and functional outcomes, allowing a comprehensive assessment of intervention effectiveness. Furthermore, consistently high adherence and retention rates across studies provided valuable evidence regarding the feasibility and acceptability of exercise interventions in this population. The inclusion of studies from multiple countries also enhances the applicability of the findings across diverse clinical settings.

Several limitations should be acknowledged. Considerable heterogeneity existed across studies with respect to exercise modality, intensity, timing relative to treatment, and outcome assessment, precluding quantitative synthesis. Most studies enrolled relatively small sample sizes, limiting statistical power and reducing generalizability to patients with advanced, recurrent, or platinum-resistant disease. In addition, patient-reported outcomes were often assessed without blinding, introducing potential reporting bias. Finally, long-term outcomes such as recurrence, progression-free survival, and overall survival were rarely evaluated, limiting conclusions regarding the sustained impact of exercise interventions.

Future implications

Future research should prioritize large, adequately powered randomized controlled trials using standardized exercise protocols and outcome measures. Particular attention should be given to determining the optimal exercise type, intensity, and timing during chemotherapy, as well as validating the potential neuroprotective effects observed in this review. Further investigation of home-based and digitally delivered exercise programs may improve accessibility and facilitate implementation in resource-limited settings. Interestingly, 78.5% of people who exercised at home continued to do so. We should look more into exercise programs people can do at home or online. Ovarian cancer patients can benefit from cancer exercise programs like these.

## Conclusions

This systematic review shows that physical activity and lifestyle changes during and after chemotherapy for ovarian cancer are very achievable (70%-100% retention and 81%-92% adherence). All six trials found that quality of life was sustained or improved, with five reporting less fatigue and three reporting improved physical function and muscle strength. Higher-intensity exercise was associated with a 50% lower risk of chemotherapy-induced neurotoxicity, although further research is needed. Taken together, the available evidence supports consideration of structured exercise interventions as an adjunct to standard ovarian cancer care. While further high-quality studies are needed to establish optimal exercise prescriptions and confirm long-term benefits, current findings indicate that exercise is feasible, safe, and potentially beneficial for improving patient-centered outcomes during and after chemotherapy.

## Appendices

Appendix 1

**Table 4:** Supplementary materials

Search details			
Pubmed ("Ovarian Neoplasms"[MeSH] OR "ovarian cancer"[tiab] OR "ovarian carcinoma"[tiab] OR "epithelial ovarian cancer"[tiab] OR "ovarian epithelial carcinoma"[tiab]) AND ("Motor Activity"[MeSH] OR "Exercise"[MeSH] OR "Exercise Therapy"[MeSH] OR "exercise"[tiab] OR "physical activity"[tiab] OR "aerobic exercise"[tiab] OR "resistance training"[tiab] OR "walking"[tiab] OR "rehabilitation"[MeSH] OR "rehabilitation"[tiab] OR "lifestyle intervention"[tiab]) AND ("Antineoplastic Agents"[MeSH] OR "Drug Therapy"[MeSH] OR "chemotherapy"[tiab] OR "chemotherapeutic"[tiab] OR "platinum-based chemotherapy"[tiab]) AND ("Fatigue"[MeSH] OR "fatigue"[tiab] OR "quality of life"[tiab] OR "QoL"[tiab] OR "physical function"[tiab] OR "treatment tolerance"[tiab] OR "neurotoxicity"[tiab] OR "muscle strength"[tiab]) Filters applied: Humans; English language.	('ovarian cancer'/exp OR 'ovarian carcinoma' OR 'ovarian neoplasm' OR 'epithelial ovarian cancer') AND ('physical activity'/exp OR exercise OR 'aerobic exercise' OR 'resistance training' OR walking OR rehabilitation OR 'lifestyle intervention') AND ('chemotherapy'/exp OR chemotherapy OR 'antineoplastic agent') AND (fatigue OR 'quality of life' OR 'physical function' OR neurotoxicity OR 'treatment tolerance' OR 'muscle strength')	( TITLE-ABS-KEY ( "ovarian cancer" OR "ovarian carcinoma" OR "epithelial ovarian cancer" ) AND TITLE-ABS-KEY ( "physical activity" OR exercise OR "aerobic exercise" OR "resistance training" OR walking OR rehabilitation OR "lifestyle intervention" ) AND TITLE-ABS-KEY ( chemotherapy OR "antineoplastic therapy" ) AND TITLE-ABS-KEY ( fatigue OR "quality of life" OR "physical function" OR neurotoxicity OR "treatment tolerance" ) )	(ovarian cancer OR ovarian carcinoma OR epithelial ovarian cancer) AND (exercise OR "physical activity" OR "aerobic training" OR "resistance training" OR walking OR rehabilitation OR "lifestyle intervention") AND (chemotherapy OR antineoplas
